# Fluoride Exposure from Ready-To-Drink Coffee Consumption

**DOI:** 10.3390/life12101615

**Published:** 2022-10-16

**Authors:** Samuel Alejandro-Vega, Daniel Suárez-Marichal, Daniel Niebla-Canelo, Ángel J. Gutiérrez-Fernández, Carmen Rubio-Armendáriz, Arturo Hardisson, Soraya Paz-Montelongo

**Affiliations:** 1 Área de Toxicología, Universidad de La Laguna, 38071 La Laguna, Tenerife, Islas Canarias, Spain; 2 Grupo Interuniversitario de Toxicología Alimentaria y Ambiental, Universidad de La Laguna, 38071 La Laguna, Tenerife, Islas Canarias, Spain

**Keywords:** fluoride, coffee, dietary intake assessment, human exposure, toxic risk

## Abstract

Fluoride is present in various foods ingested daily. It has been demonstrated that the intake of high concentrations of fluoride, both in adults and children, can cause pathologies, among which dental fluorosis, osteoporosis and damage to the central nervous system stand out. The objective of this study was to determine the fluoride concentrations in 60 samples of ready-to-drink cold brewed coffee of different brands and types (expresso, cappuccino, macchiato and decaffeinated) by the fluoride ion-selective potentiometric method. A statistical analysis was also performed to discern the existence of differences between these categories. The highest fluoride concentration (1.465 mg/L) was found in espresso coffee followed by *Macchiato* (1.254 mg/L). Decaffeinated coffee is the one that presented the lowest fluoride concentration with 0.845 mg/L. The risk assessment was conducted considering different consumption scenarios (250, 500 and 750 mL/day). The UL (upper level) established by the EFSA (European Food Safety Authority) at 7 mg/day was used. The consumption of three servings poses no health risk; however, it confers a significant value of fluoride contribution to the diet.

## 1. Introduction

Fluorine is a highly electronegative element belonging to the halogen group, which has the highest reactivity in the periodic table. It is one of the most abundant elements in the earth’s crust, as it forms part of various minerals mainly from volcanic origin. For this reason, regions with volcanic soil tend to be associated with a higher fluoride content in their groundwater [1].

Water represents the main source of fluoride, which is present in various beverages consumed on a daily basis, such as coffee. According to the European Food Safety Authority (EFSA) [2,3], approximately 75% of the fluoride ingested comes from the consumption of water and beverages prepared with it, containing more than 0.3 mg/L of fluoride. Furthermore, it should be noted that studies by Wolska et al. [4] show that coffee can contain significant concentrations of fluoride, for example, in Turkish coffee, where the authors reported an average concentration of 0.50 mg/L, or espresso with an average concentration of less than 0.20 mg/L. Therefore, it has been shown that the germination process, the irrigation of the plant itself and other factors can influence and contribute to the incorporation of this ion into coffee [2,5].

It is also worth considering the increase in fluoride in the environment and thus in food. Fluoride can be released from human activities, especially in countries with high industrial activity or agricultural activities [6]. On the other hand, natural sources of fluoride are abundant, especially in regions with recent volcanic activity [7].

According to EFSA, fluoride is not an essential nutrient and therefore no recommended intake can be given for the proper development of physiological functions [2]. The harmful effects on health have been reported, as exposure to high concentrations of this ion over time produces different pathologies, including dental fluorosis, in which brownish stains are formed due to the hypomineralization of the enamel, thus losing its protective capacity against bacteria, which leads to the appearance of caries [8,9]. On the other hand, in skeletal fluorosis, fluoride accumulates in bone tissue, causing an increase in bone density. Fluoride damage can be even more severe, with reported damage to soft tissues such as the kidney, liver, brain and even reproductive organs [1,10,11,12,13,14,15,16,17]. Several studies confirm the relationship between the consumption of water supplies with high fluoride concentrations during pregnancy and the birth of babies with lower IQs [18,19].

However, considering the role of fluoride in terms of reducing the occurrence of dental caries or increasing the hardness of tooth enamel [20], the EFSA has established adequate intake values. Table 1 lists the reference values for adequate intake and maximum intake level for fluoride established by EFSA [3]. 

As these products have been widely consumed in recent years, and coffee is a beverage that is notable for its fluoride content, it is necessary to assess the intake of fluoride from the consumption of ready-to-drink coffee beverages. The objectives of this study were (1) to determine the fluoride concentration in different types and brands of ready-to-drink coffee beverages and (2) to assess the dietary fluoride exposure due to ready-to-drink coffee intake.

## 2. Materials and Methods

### Samples and Solutions

Table 2 lists the characteristics of the samples analyzed. A total of 60 samples of different Coffee types were acquired (20 espresso samples, 10 decaffeinated samples, 20 cappuccino samples and 10 macchiato samples).

The treatment and determination of fluoride require the prior preparation of different solutions, which are listed below:

- Orthophosphoric acid conditioning solution (0.75 M): 51 mL of 85% pure orthophosphoric acid (H_3_PO_4_) (Sigma Aldrich, Germany) is diluted in 1 L of Mili-Q distilled water (Milli-Q Gradient A10, Millipore, MA, USA).
- 10^−1^ M fluoride standard solution: 0.428 g of sodium fluoride (NaF) (HoneyBell Fluka, Germany) of 98% purity, previously dried in an oven for 24 h at 120 °C (Nabertherm, Germany), is dissolved in 100 mL of Mili-Q distilled water.
- Serial fluoride dilutions: 10^−1^ M, 10^−2^ M, 10^−3^ M, 10^−4^ M and 10^−5^ M dilutions are prepared from the stock solution.

## 3. Fluoride Determination

Potential determination was carried out using the potentiometer model HACH sensION-MM340 (HACH, Düsseldorf, Germany) and a fluoride ion selective electrode (ISE) (HACH ISE F-9655C, Düsseldorf, Germany). 

The instrumental parameters are: range (0.01 mg/L to 19,000 mg/L), pH range (4–8), linear range (0.1 mg/L to 19,000 mg/L fluoride), slope (59 mV/pF), and operating temperature (5–50 °C). Possible interferences: Fe^3+^ and Al^3+^, removed by the conditioning solution.

The potentials of the serial fluoride solutions were measured and the calibration line was obtained. The repeatability of the method was checked through daily measurements of the calibration curve, obtaining linearity coefficients higher than 0.9990 (r2).

Once the calibration line was obtained, the samples were prepared prior to the power measurement. Briefly, 25 mL of each ready-to-drink cold coffee sample, previously homogenized, was introduced and 5 mL of the 0.75 M orthophosphoric acid solution (H3PO4) was added in a polypropylene cup [21]. The potential (mV) is measured in triplicate and under constant stirring. From the potentials obtained (mV) and the calibration line prepared, the fluoride concentrations of each sample are interpolated [1]. 

### 3.1. Method Quality Control and Validation

The precision of the method was evaluated under reproducibility conditions using the standard additions method. The fluoride concentration in the samples was determined and subsequently, a known amount of fluoride was added. This process was repeated 15 times/day on 3 alternate days. 

The repeatability values in terms of relative standard deviation (RSD) were 2.40% with a reproducibility of 3.20%. The sample recovery rate was 99.5% with an RSD of 0.55%. The values obtained were satisfactory and the recovery percentages were high. 

### 3.2. Statistical Analysis

Statistical analysis was carried out using GraphPad Prism 8.0.1 software (GraphPad Software Inc., San Diego, CA, USA). 

The aim of the statistical analysis was to detect the existence of significant differences (*p* < 0.05) in fluoride content between coffee types (espresso, cappuccino, macchiato and decaffeinated) and between brands (A, B, C and D). Anderson-Darling, D’Agostino and Pearson and Shapiro–Wilk normality tests were applied [22]. It was found that the data set did not follow a normal distribution, so non-parametric tests were used. The non-parametric tests used were the Kruskal–Wallis and Kolmogorov–Smirnov [23,24].

### 3.3. Dietary Intake Assessment

The dietary intake and subsequent toxic risk assessment is based on first obtaining the Estimated Daily Intake (EDI) (Equation (1)) and then the percentage contribution (Equation (2)) considering the Adequate Intake (AI) and Upper Level (UL) value for fluoride established by EFSA [3].

(1) EDI=Fluoride concentration (mg/L)×Daily consumption (L/day)

(2) $$\mathrm{Contribution}\left(\%\right)=\frac{\mathrm{EDI}\;(\mathrm{mg}/\mathrm{day})}{\mathrm{Reference}\;\mathrm{value}\;(\mathrm{mg}/\mathrm{day})}\times 100$$

## 4. Results and Discussion

### 4.1. Statistical Analysis Results

A non-normal distribution was found; therefore, Kruskal–Wallis non-parametric tests were used. Statistical analysis detected significant differences (*p* < 0.05) in cappuccino vs. espresso (*p* = 0.033) and espresso vs. decaffeinated (*p* = 0.0053). In the brand study, significant differences were found between brands A vs. C (*p* = 0.0005), B vs. C (*p* = 0.0001) and C vs. D (*p* < 0.0001). These differences are due to the difference in the fluoride content of their ingredients as the water and the origin of the coffee bean could be important contributors if the water supply or the soil from the raw ingredients have a high fluoride content.

### 4.2. Fluoride Concentration in Ready-To-Drink Coffee

Table 3 shows the average fluoride concentrations (mg/L) recorded in the samples both by coffee types and by brands.

In terms of coffee types, espresso coffee (1.465 mg/L) stands out as having the highest average fluoride value. This may be due to the higher proportion of coffee in this type of beverage. Meanwhile, decaffeinated coffee (0.845 mg/L) has the lowest concentration of fluoride, which may be due to the process by which the caffeine content of the coffee is reduced, which may be capable of dragging and eliminating part of the fluoride it contains.

The highest average fluoride concentrations in the samples by brand were found in brand B coffees (1.425 mg/L), with brand D (1.377 mg/L) and A (1.341 mg/L) also standing out. These recorded fluoride concentrations may be due to multiple factors such as the high fluoride content of the raw material or the presence of an ingredient that may contain high concentrations of this anion. The coffee samples of brand C stand out for their low fluoride content, registering an average concentration of 0.812 mg/L. This may be due to the type of coffee used (Arabica coffee), the use of water with a high fluoride content, or even the absorption of fluorides by the coffee plant itself. However, it is worth noting that brand C has the highest fluoride value in one of the samples of this brand (2.883 mg/L), which shows the great variability in the content of this anion even in the same brand. This fact highlights the need to control the fluoride content in the raw materials used for the production of this type of product, as it directly influences the fluoride content in the final product.

Coffee is one of the most widely consumed beverages in the world, along with tea. In studies conducted by other authors who determined the fluoride content in coffee, widely varying concentrations are reported. Thus, Olechno et al. [25] report fluoride concentrations in roasted arabica coffee (7.5 µg/100 mL of coffee) and in green coffee (14 and 50 µg/100 mL of coffee), which, as can be seen, vary from one type of coffee to another. Studies carried out by Wolska et al. [4] recorded fluoride concentrations in coffee ranging from 0.013 to 0.502 mg/L, concluding that the preparation conditions and type of coffee can affect the concentration. Satou et al. [26] also analyzed the fluoride content in coffee, obtaining concentrations of 0.03–0.15 mg/L and 0.04–0.64 mg/L. In ready-to-drink coffees, the process by which the coffee was obtained is not indicated.

As for tea, high concentrations of fluoride are reflected, with the highest concentration in decaffeinated tea from India (4.32 mg/L) and the lowest in green tea from China (0.63 mg/L), depending on the type of tea and the water used in its preparation. Therefore, not only coffee is a source of fluoride, but tea also contributes to fluoride intake [1,27,28,29,30,31]

### 4.3. Dietary Intake Assessment

The assessment of dietary intake has been established considering three possible consumption scenarios, corresponding to 1, 2 and 3 servings of the product (0.25, 0.50 and 0.75 L/day) (Table 4). A population older than 18 was considered since these products are not consumed by underage people.

Coffee consumption by type provides estimated daily intakes of 0.211–0.366 mg/day for a single daily serving (250 mL). When consumption increases to three servings per day (750 mL), fluoride intakes can reach values of 0.634–1.10 mg/day. It should be noted that the type of coffee that provides the most fluoride is espresso. In terms of fluoride intake by brand, brand B has the highest contribution with estimated daily intakes of 0.356–1.07 mg/day from 1 to 3 servings.

Considering the EFSA UL (upper level) value of 7 mg/day, this gives a contribution rate of 3.02–5.23% in the case of the consumption of a single serving. When consumption increases to three servings per day the percentage contribution rises to 9.05–15.7%, with the highest percentage contribution of fluoride in espresso coffee. The highest percentages of contribution to fluoride intake in terms of brands are due to the consumption of the brand B product (5.09–15.3% from 1 to 3 portions, respectively).

The values for the contribution to the dietary intake of fluoride from the consumption of these products do not represent a health risk, but it is necessary to take into account that it is a single food and that the overall dietary intake of fluoride is likely to be higher. It is necessary to study the overall fluoride intake in the adult population to avoid risks, as there are populations living in places with high fluoride content in the water supply, such as the northern regions of the island of Tenerife (Canary Islands, Spain) or countries such as India, China, Pakistan, Argentina and some regions of the United States [32,33,34,35].

### 4.4. Consumption Recommendations

Consumption recommendations should be adapted to the individual consumption of each consumer. According to data from the latest Spanish Annual Food Consumption Report 2021 [36], the annual consumption of the coffee and infusions group is 1.94 kg/person/year, which is a high consumption by the Spanish population. Although there are no data referring to these ready-to-drink coffee products, we cannot ignore the fact that its growing presence in markets is due to its high consumption.

Taking into account the results obtained in the present study, considering 1 to 3 servings per day, it is necessary to warn the population that a high consumption could infer a high intake of fluoride.

The percentages of the contribution to the UL shown above reflect the impact of fluoride in the diet, where it can be seen that the intake of more than three ready-to-drink cold coffees in adults may entail a health risk as it is a single product and, therefore, it is necessary to know the fluoride content in other foods and in the water supply in order to establish consumption recommendations for this type of product. However, in general terms, we can establish certain guidelines for responsible consumption (Table 5).

## 5. Conclusions

The fluoride content of ready-to-drink cold coffee beverages of different types (cappuccino, espresso, decaffeinated and macchiato) of four different commercial brands was determined. Fluoride concentrations were obtained using the fluoride ion selective potentiometric technique.

With regard to the type of coffee, espresso is the one with the highest average concentration. In the study by brand, brand B has the highest fluoride concentration. Significant differences were found between cappuccino and espresso, and between espresso and decaffeinated coffee, which shows that the process by which coffee is obtained and prepared influences the fluoride content in the final product, among other possible factors.

The dietary fluoride intake study shows estimated daily intake values that do not exceed the UL value for adults set by EFSA, but nevertheless, as this is a single product, it is necessary to assess the overall fluoride intake and especially whether consumers are exposed to high concentrations of fluoride in the water supply.

## Figures and Tables

**Table 1:** Values of AI (adequate intake) and UL (upper intake level).

Stage	Age	Gender	AI (mg/day)	UL (mg/day)
Adults	≥18 years	Men	3.4	7
		Women	2.9	
Pregnant women				
Lactating women				

**Table 2:** Description of the analyzed samples.

Brand	Coffee Type	No. Samples	Composition Reported Products’ Label
A	Espresso	5	Semi-skimmed milk (76.4%), coffee (18.4%) (water, soluble Arabica coffee (1.5%)), sugar and stabilizers.
	Cappuccino	5	Semi-skimmed milk (79%), coffee (14%) (water, soluble Arabica coffee (1%)), sugar, defatted cocoa powder (0.35%) and stabilizers.
	Macchiato	5	Whole milk (79%), coffee (15%) (water, soluble Arabica coffee (0.95%)), sugar and stabilizers.
B	Espresso	5	Semi-skimmed milk (77%), coffee (18%), sugar and stabilizers.
	Decaffeinated	5	Semi-skimmed milk (73%), decaffeinated coffee (17%), cream (5%), sugar and stabilizers.
	Cappuccino	5	Semi-skimmed milk (77%), coffee (17%), sugar, caramelized sugar syrup, cocoa powder and stabilizers.
C	Espresso	5	Semi-skimmed milk (80%), Arabica coffee (18%) and sugar.
	Decaffeinated	5	Whole milk (80%), Arabica coffee (16%) and sugar.
	Cappuccino	5	Semi-skimmed milk (80%), Arabica coffee (15%), sugar and cocoa powder (0.2%).
D	Espresso	5	Skimmed milk (85%), cream, sugar, skimmed milk powder and soluble coffee (1.4%).
	Cappuccino	5	Skimmed milk (85%), cream, sugar, skimmed milk powder, soluble coffee (1%) and cocoa powder (0.03).
	Macchiato	5	Skimmed milk (84%), cream, sugar, skimmed milk powder and soluble coffee (1%).

**Table 3:** Mean concentrations, standard deviation, minimum and maximum values of the ready-to-eat coffee.

Coffee Type	Cappuccino	Decaffeinate	Espresso	Macchiato
Mean concentration (mg/L)	1.167	0.845	1.465	1.254
Min. value (mg/L)	0.853	0.362	0.422	0.866
Max. value (mg/L)	1.550	1.461	2.883	1.675
Standard deviation (SD) (mg/L)	0.209	0.486	0.641	0.329
**Brands**	**A**	**B**	**C**	**D**
Mean concentration (mg/L)	1.341	1.425	0.812	1.377
Min. value (mg/L)	0.866	0.957	0.362	1.078
Max. value (mg/L)	2.046	2.751	2.883	1.675
Standard deviation (SD) (mg/L)	0.412	0.428	0.642	0.186

**Table 4:** Values of EDI (mg/L) and contribution percentages to the UL set by the EFSA (2006).

EDI (mg/L)												
Coffee type	Cappuccino			Decaffeinate			Espresso			Macchiato		
	250 mL/day	500 mL/day	750 mL/day	250 mL/day	500 mL/day	750 mL/day	250 mL/day	500 mL/day	750 mL/day	250 mL/day	500 mL/day	750 mL/day
	0.292	0.584	0.875	0.211	0.423	0.634	0.366	0.733	1.10	0.314	0.627	0.941
Brand	A			B			C			D		
	250 mL/day	500 mL/day	750 mL/day	250 mL/day	500 mL/day	750 mL/day	250 mL/day	500 mL/day	750 mL/day	250 mL/day	500 mL/day	750 mL/day
	0.335	0.671	1.01	0.356	0.713	1.07	0.203	0.406	0.609	0.344	0.689	1.03
Contribution Percentages (%) to the UL												
Coffee type	Cappuccino			Decaffeinate			Espresso			Macchiato		
	250 mL/day	500 mL/day	750 mL/day	250 mL/day	500 mL/day	750 mL/day	250 mL/day	500 mL/day	750 mL/day	250 mL/day	500 mL/day	750 mL/day
	4.17	8.34	12.5	3.02	6.04	9.05	5.23	10.5	15.7	4.48	8.96	13.4
Brand	A			B			C			D		
	250 mL/day	500 mL/day	750 mL/day	250 mL/day	500 mL/day	750 mL/day	250 mL/day	500 mL/day	750 mL/day	250 mL/day	500 mL/day	750 mL/day
	4.79	9.58	14.4	5.09	10.2	15.3	2.90	5.80	8.70	4.92	9.84	14.8
UL set by EFSA for adults regardless of gender = 7 mg of fluoride/day												

**Table 5:** Recommendations for consumption of ready-to-drink cold brew coffee considering the fluoride content of the water supply.

Ready-To-Drink Cold Coffee (Servings)	Volume (mL)	Fluoride in Supply Water (mg/L)	Risk? Yes/No
1	250	<1.5	NO
		1.5–3	NO
		>3	YES
2	500	<1.5	NO
		1.5–3	YES
		>3	YES
3	750	<1.5	NO
		1.5–3	YES
		>3	YES

## Data Availability

Data are contained within the article.
